# On the beauty of sadness: it’s okay to say, I am sad, thank you

**DOI:** 10.1080/19420889.2023.2211424

**Published:** 2023-05-12

**Authors:** Tobore Onojighofia Tobore

**Affiliations:** Independent Scholar, Yardley, Pennsylvania, USA

**Keywords:** Depression, diminishing returns, emotions, evolution, gene-environment interaction, genetics, grade inflation, greetings, happiness, happy birthday, happy holidays, helicopter parenting, heritability, low mood, materialism, narcissism and happiness, negative emotions, negative moods, post-traumatic growth, pursuit of happiness: sadness rebranding, resilience, sadness

## Abstract

We live in times when our culture is obsessed with happiness. The value of almost every aspect of our lives is increasingly judged in terms of their contribution to our happiness. Happiness has become the ultimate goal by which values and priorities are constructed and the only thing for which any action in pursuit of does not require justification. In contrast, sadness is increasingly abnormalized and pathologized. In this paper, an effort is made to counteract the narrative that sadness, a critical aspect of human life is abnormal or a pathological condition. The evolutionary benefits of sadness and its place in human flourishing are discussed. A rebranding of sadness is proposed that emphasizes the free expression of sadness in everyday greetings to remove it from its current negative state and promote many of its benefits including post-traumatic growth and resilience.

## Introduction

Happiness is a key ingredient of well-being, and most people desire it more than anything else [1]. Happy people tend to have more friends, richer social interactions and social support, higher quality of work, increased productivity, and higher income [2]. There have been different definitions of happiness based on philosophical arguments and human search for wisdom [3]. However, it has traditionally been described in two different forms: hedonic and eudaimonic [4]. Eudaimonic happiness or a well-lived life [5] occurs when people are fully engaged, and their life’s activities are in line with their true selves and values [4]. Hedonic happiness is often equated with immediate pleasure or joy, the absence of negative affect, psychophysical well-being, and a high degree of life satisfaction [6–8].

In recent years, the prevailing understanding of happiness has been the hedonic version [3]. We live in times when our culture is obsessed with this version of happiness. Evidence from a recent YouGov research indicates that over 80% of Americans wished to be happy [9]. Happiness has become the ultimate goal by which values and priorities are constructed and the only thing for which any action in pursuit of does not require justification [10]. The value of almost every aspect of our lives from relationships (friends, partners, and spouse), jobs, body, diet, etc. are increasingly judged in terms of their contribution to our happiness. Anything or anyone not contributing to one’s happiness is viewed as undesirable and unworthy of accommodation. In line with this happiness zeitgeist [11], information posted on social media by people are often socially desirable that shows them in positive or happy moments [12,13]. Research on happiness or subjective well-being largely studied in the sphere of positive psychology has increased in recent years [14]. Bookstores are full of self-help materials selling in the millions that promote the endless benefits of happiness [2]. In addition, demand for life coaches, social media influencers promoting wellness and positivity as well as motivational speakers has increased in recent years [11].

Distinguished as beneath depression, states of low mood or negative emotions described in the paper as sadness is an adaptive emotion and a mild temporary mood state characterized by low physiological arousal [15]. Sadness may also be described as a psychological pain typically associated with feelings of distress, loneliness, anguish, grief, etc [16]. Sadness provides significant benefits in human lives including promoting optimal performance in social judgments, memory, and motivation [17] and allowing humans to cope with losses and life’s difficulties [18]. In this environment where happiness is greatly sought after, sadness has become a great evil to be avoided. Indeed, evidence suggests that emotions associated with sadness which are known to be a critical part of the human condition are increasingly pathologized as disorders [19,20]. Sadness even from loss, true hardship, and discrimination is described as abnormal and personal failure which may lead to self-blame [21,22]. Also, this happiness zeitgeist that abnormalizes sadness has affected our treatment of mental health. Indeed, over-treatment, excessive medicalization, and ever broader definition of mental health conditions to the point where normal human life experiences are considered to be illnesses that require treatment is a pervasive and troubling problem in the field of mental health [23–26]. This happiness zeitgeist has also negatively impacted modern parenting and events in our educational institutions with pernicious national and political implications.

The objective of this paper is to discuss the importance and benefits of sadness in human lives, the dangers of treating it as a state to be avoided as well as the need for us to express it more openly for a more flourishing human experience.

## Human condition, evolution, and happiness

Evidence suggests that happiness or desirable mental experiences have been of interest to humans in different cultures and throughout history [27]. Happiness is believed from an evolutionary perspective to be useful in promoting human reproduction and survival [28–31]. Winners of competition compared to losers experience an endocrinal and hedonic boost [32]. Studies indicate a key role of heritability/genetics and gene environment in happiness [33–36]. Happiness is associated with positive impact, success, good health, and good health correlates [3,37,38], greater life satisfaction [39–42], and cognitive flexibility [43].

However, from an evolutionary standpoint, perpetual happiness is impossible and is believed to likely have been evolutionarily non-beneficial or maladaptive [44]. Indeed, humans have evolved adaptations that interfere with perpetual happiness or the pursuit of happiness [29]. These adaptations include subjective distress (e.g., psychological pain, anxiety, fear, anger) from events that occur in our lives and environment, competitive adaptations (e.g., jealousy, envy) that produce psychological pain, as well as big differences between our ancestral communities and modern societies [29,45]. Furthermore, human experience of pleasure and satisfaction yields diminishing positive returns. The first banana you eat tastes so good, but after several, the satisfaction derived from each additional banana lessens because you grow tired of the taste. You may sleep with the most attractive person in the world, but over time, your sexual interest wanes [46]. Your greatest sexual encounter is soon replaced by another, and soon, everything is a passing memory.

These diminishing returns also extend to extrinsic achievements (e.g., wealth, power, educational attainment, work). Some people think that prioritizing extrinsic achievements will bring happiness, but evidence suggests otherwise [47,48]. Our modern consumer-focused society is flooded with materialistic communications (from marketing, social media wealth flaunting, rags-to-riches movie stories, and documentary series, to music videos and reality television shows depicting opulent lifestyles) that connect success, high self-esteem, social recognition, and happiness to wealth and consumption [49]. This culture linking social status to success and happiness also promotes narcissism [50,51], which is believed to be on the rise, especially in young people [52,53]. However, research shows that money can buy a person some level of happiness, but above a certain threshold, more money does not translate into more happiness [54]. Many people get richer during their lifetimes, but this does not necessarily translate into greater happiness over their lifetime.

Our competitive nature makes life an onward movement from interest to interest, from one desire to another, making constant happiness impossible. As one set of aspirations is realized, we soon move on from it in pursuit of bigger and more exorbitant aspirations, which we hope will bring us happiness [55]. For example, the pleasure of buying a new luxury car is quickly neutralized by the news that a friend just purchased a more expensive and luxurious car. The sense of personal satisfaction gained from landing your dream job quickly dissipates when you hear that a close friend was promoted to a more senior position at a larger corporation with better benefits and double the salary.

Wellness gurus and self-help coaches repeat the platitude that happiness is always within grasp if you just simply focus on the positives and stamp out “negative” thoughts [21,22]. They encourage cutting off anything or anyone that interferes with happiness goals. The point missed is that the world is a dynamic place and happiness is not always within grasp. Encouraging people to feign happiness amid sadness or treat sadness as nonexistent is tantamount to telling them to deny or evade reality. What happens when the individual in fervent pursuit of happiness becomes the source of the sadness because they fell on hard times or became chronically ill? Suppose they have no true friends or family around them because they have cut off every loved one or previously cut off friends and family chose to give them a dose of their own medicine by ignoring them.

Several key factors including individual and collective civic engagement, trust, and social capital, as defined by the strength of family, community, and workplace ties, are strongly linked to happiness [54]. Also, self-transcendence, selflessness, and generosity are strongly associated with increased happiness [48,56–59]. However, although family and social connections are important, a person cannot choose their biological family. Many people find themselves born into toxic and dysfunctional families that they want little to nothing to do with. Even good families could grow dysfunctional over time. There is no guarantee of a happy marriage or well-adjusted children, even with one’s best efforts, and there is no guarantee that your child will not die before you. Friends can betray, even with all the kindness shown to them, and generosity increases the expectation of reciprocity, which is not guaranteed. Unrequited generosity elicits great psychological pain. One could give their talents and time to a company and develop strong ties with colleagues, but at the first sign of problems, they are laid off. Companies exist to make money, and an employee is only as valuable as their ability to continually contribute to the company’s growth and development. A change in company priorities can quickly test the tie between a company and its employee, regardless of previous performance and contributions. Of all the dangers in this world, there is none greater than for an individual to base their present or future happiness, success, and livelihood on the vagaries of someone else’s interests, intentions, or behavior. The recent Silicon Valley layoffs due to the challenging economic situation is a pertinent case in point [60,61] where employees many of which have worked for decades at these companies were unceremoniously laid off.

Happiness has a role to play in human flourishing as evidenced by its evolutionary benefits. However, evolutionary adaptations and human dynamic life experiences interfere with perpetual happiness indicating that there is a limit to the benefits of happiness and that happiness alone is not sufficient for human flourishing.

## Sadness, a critical aspect of the human condition

Evolutionary theories suggest that adaptations that lead to sadness have been largely shaped by the functional demands of our ancestral environment [17,62]. Neuroimaging studies on humans have shown that sadness is a basic emotion, and this is supported by activity in certain brain regions including the subgenual anterior cingulate, insular, amygdalar, and orbitofrontal activation [63,64]. Multiple lines of evidence indicate that sadness is associated with lower cortical activation over evolutionarily primitive systems of the brain [16]. The evolutionary foundations and PANIC/GRIEF system of sadness have been extensively described [65,66]. Also, epigenetic or genotypic variations have been linked to negative emotions [16]. Indeed, DNA methylation in certain genes and their associated neural processes are linked to sadness [16].

Sadness is critical for post-traumatic growth (PTG) [67,68] which includes a greater appreciation for life or positive reevaluation of life, better relationships, appreciation of newer possibilities, increased personal strength, and a greater sense of spiritual development from coping with a traumatic experience [69]. Research indicates that there is gene-environment interaction that promotes PTG [70]. The SARS epidemic on the general public in Hong Kong brought some positive impacts including lifestyle changes, social/family support, and increased mental health awareness [71]. Being diagnosed with a life-threatening disease elicits psychosocial and spiritual reflection that can lead a person to reorganize their values and assessment of life [72]. Indeed, cancer patients feel hgenerally more satisfied with life, likely due to their altered expectations about the future and their increased ability to value the simple aspects of everyday life [73].

Furthermore, sadness is not just an essential and normal component of human lives but an integral piece of a flourishing life [19]. Several virtues of sadness have been identified including serving as a protective mechanism from harmful situations, means of conserving resources and energy, increasing the accuracy of judgment and perception, and as means of expressing care, compassion, and love [19]. Sadness reduces judgmental errors [74]. It strengthens and promotes better eyewitness memory [75] and helps people to connect by creating a sense of shared values and togetherness [76]. It is an important architect of cognitive change, by allowing for self-reflection of goals and beliefs [77]. Sadness or negative moods have also been found to be associated with emotional and cognitive creativity [78]. Indeed, rumination and reflection are positively correlated with negative mood, and during the outbreak of COVID-19, evidence suggests that negative mood modulated creative ideation via the mediation of reflection and rumination [78]. Negative mood promotes more self-related thoughts and makes individuals more likely to engage in ruminative and reflective thinking [79].

Also, sadness promotes resilience [80,81]. Adverse life experiences in moderation may promote resilience, with resulting benefits for well-being and mental health [80]. Research indicates the experience of some lifetime adversity predicts lower functional impairment, lower global distress, higher life satisfaction over time, and fewer posttraumatic stress symptoms [80]. Similarly, a moderate number of adverse life experiences is associated with more positive psychophysiological responses and less negative responses to pain [81].

## Discussion

Many people desire happiness in their lives [1] and happiness is associated with significant benefits. However, just as food is important for human flourishing but can be harmful, happiness is not always good [11]. Our current zeitgeist which places great value on happiness may be a risk factor for depression [82]. Obsessively pursuing happiness or having happiness as the desired emotional endpoint as a goal is associated with loneliness, poor psychological well-being, and life satisfaction [83–86]. Furthermore, putting great importance on experiencing happiness may result in maladaptive emotion control [87], persistent self-mood monitoring, emotion regulatory efforts, and the denial or evasion of current unpleasant emotions considered irreconcilable with the desired positive mood [11,85].

The elevation of happiness as the most important thing in life in modern times contributes to the increase of helicopter parenting. Helicopter parenting is a phenomenon believed to have started rising since 1985 [88] that entails the over-involvement (child overprotectiveness in a controlling manner) of parents in their children’s lives [89,90]. It has become a widespread cultural norm in all social classes [91]. Helicopter parenting is linked to the need for parents to shield their children from sadness and one of its hallmarks is the tendency for parents to jump and extricate their children from sad and unpleasant experiences [92]. Helicopter parenting is associated with harmful outcomes for children including negative psychological well-being, anxiety/depression, and the consumption of prescription opioids recreationally [89,93,94]. It is associated with maladaptive behavior toward managing distressing experiences [95]. Indeed, research indicates that helicopter parenting is linked to the development of an avoidant interaction response pattern to stressful situations [95–97], children’s increased sense of entitlement, and maladaptive academic motivations [98].

The abnormalizing of sadness has also affected students’ behavior in higher learning institutions. Grade inflation is well documented over the last 30 years [99] at private and publication higher institutions of learning in the United States [100]. Happiness is associated with students ‘grades [101,102] and one factor that contributes to this grade inflation is the need by higher learning institutions to make students, who are increasingly treated as customers, happy [100]. Increasingly, higher institutions kowtow and placate students many of which are products of helicopter parenting for every grievance and give priority to their feelings including who speaks on campus and the teacher/professors they prefer. The recent firing of a college professor whose students complained about their grades and the class rigor is a pertinent case in point [103]. The use of student evaluation to assess teaching effectiveness is a tool that students use to punish perceived erring teachers, and this contributes to grade inflation [104]. So, a confluence of factors including students’ inability to handle the distress of poor grades, teachers who are concerned about negative student evaluation if they grade strictly on merit or impose rigor in their teaching, colleges who are concerned about student enrollment numbers, and brand image if students complain and the abnormalization of academic stress, rigor, and poor grades by linking it to mental well-being [105] are key contributors to the issue of grade inflation in recent years. If students are constantly shielded from everything or anything that causes disappointment and pain, how can they grow psychologically or deal with pain in the real world? These developments have negative implications for social, political, and national development.

Most people spend significant moments in their day in negative moods. Research suggests that while awake most people spend almost 50% of their time thinking about something different from what they are doing [106]. Daily, the average adult spends some time lost in their thoughts ruminating about events involving friends, children, spouse, career, family, and people they barely know they might have read or heard about from the news, etc. and this mind-wandering usually makes them unhappy [106]. Since many people spend a significant portion of their lives in negative moods, abnormalizing that experience as the evil twin of happiness to be avoided is harmful to human flourishing. Sadness distinguished as beneath clinical depression is a basic emotion that plays an important role in human lives from PTG to creativity. Many human achievements are the results of people’s unhappiness with the state of affairs and not their happiness with the state of affairs.

Greeting is one of the primary functions of communication that helps connect people across cultures at a personal level. It is typically associated with expressing respect and positive emotions. Describing oneself as unhappy while exchanging greetings with people may elicit concern from the other party with an offer of help because everyone is expected to be happy or doing well. Expressing sadness may also be socially undesirable as it can paint a negative image of the person expressing it. As a step toward reversing this trend of abnormalizing and pathologizing sadness, it is important for people to freely express sadness without feeling ashamed or eliciting pity, and disgust from others. A critical predictor of PTG is sharing negative emotions [69,107]. This paper proposes that to remove sadness from its current negative state and promote PTG, resilience, and other benefits of sadness, people should express it more frequently without any negative social ramifications. Just as one can greet by saying “I am fine, thank you or I am doing well, thank you”, one should be able to express their state of sadness not just to friends and family but to anyone by saying “I am sad, thank you or I am not doing well, thank you”, as part of normal daily greeting. Instead of saying happy holidays or happy birthday, for many people dealing with sadness, wishing them resilience and PTG is likely to be more appropriate.

Furthermore, parents should be encouraged to see sadness, not as a bad thing to protect their children from but to discuss the benefits of disappointment and sadness with their children and work with them to inculcate the values and growth that come with it. Society must move away from the abnormalization of sadness and obsession with happiness. Well-meaning institutional or government actions and policies based around the idea that sadness is bad, and people deserve and should be happy all the time are counterproductive and are likely to have pernicious consequences.

The goal of this paper is not to promote sadness but to counteract the narrative that sadness, an inescapable aspect of the human experience, should be treated as the evil twin of happiness. It is not to celebrate sadness, encourage people to pursue sadness, or wish sadness for others. But to deal with sadness and show understanding for others dealing with sadness for a more flourishing life and better human experience. It is to describe the many benefits of sadness as well as the rebranding of sadness to remove it from its current negative state. Expressing negative emotions is critical to PTG and expressing sadness in everyday greetings between people will foster solidarity and a world where people are more supportive of one another.

Finally, the greetings noted in Table 1 are for people to express sadness more freely and the responses are specifically for people who expressed sadness or are known to be dealing with sadness. Although sadness is important, individual differences exist in biological sensitivity to negative experiences [108]. Also, there exists a depression continuum where sadness lies somewhere in the middle between well-being and depression [109], indicating that the line between sadness and depression appears to be nebulous. So, people dealing with sadness they cannot manage or with persistent and deep sadness should seek support and be given all the support they need.

**Table 1.** Alternative ways of daily greeting that express Sadness or greet people dealing with Sadness.

Alternative greeting involving someone asking, “How are you.”	Alternative greeting involving wishing “Happy Birthday	Alternative greeting involving wishing “Happy Holidays”
**First person**: How are you? **Second person**: I am sad, thank you. I am not doing well, thank you. I am unhappy, thank you. **First person**: Thanks for expressing. All the best with your recovery. Thanks for expressing. Wishing you resilience and post-traumatic growth.	Wishing you resilience for your birthday Wishing you post-traumatic growth for your birthday Wishing you resilience and post-traumatic growth for your birthday.	Wishing you resilience this holiday Wishing you post-traumatic growth this holiday Wishing you resilience and post-traumatic growth this holiday.

The above greetings are examples of ways of expressing and greeting people dealing with sadness. There are potentially many other ways to express sadness and greet people dealing with sadness. The examples above can be an inspiration for more ways to express sadness or appropriately greet someone dealing with sadness daily.
